# Targeting *Pseudomonas aeruginosa* resistance at the exposure frontier: a population PK/PD blueprint for ceftolozane/tazobactam continuous infusion

**DOI:** 10.1128/aac.01042-25

**Published:** 2025-09-22

**Authors:** Pier Giorgio Cojutti, Manjunath P. Pai, Milo Gatti, Matteo Rinaldi, Tommaso Tonetti, Antonio Siniscalchi, Pierluigi Viale, Federico Pea

**Affiliations:** 1 Department of Medical and Surgical Sciences, Alma Mater Studiorum, University of Bologna 9296 , Bologna, Italy; 2 Clinical Pharmacology Unit, IRCCS Azienda Ospedaliero-Universitaria di Bologna, Bologna, Italy; 3 Department of Clinical Pharmacy, College of Pharmacy, University of Michigan, Ann Arbor, Michigan, USA; 4 Infectious Diseases Unit, IRCCS Azienda Ospedaliero-Universitaria di Bologna, Bologna, Italy; 5 Anesthesiology and Intensive Care Medicine, IRCCS Azienda Ospedaliero-Universitaria di Bologna, Bologna, Italy; 6 Division of Anesthesiology, Department of Anesthesia and Intensive Care, IRCCS Azienda Ospedaliero-Universitaria di Bologna, Bologna, Italy; Providence Portland Medical Center, Portland, Oregon, USA

**Keywords:** population pharmacokinetics, ceftolozane/tazobactam, PK/PD target attainment

## Abstract

Ceftolozane/tazobactam is a key antibiotic for *Pseudomonas aeruginosa* infections. Our objective was to determine whether continuous infusion (CI) of ceftolozane/tazobactam can achieve aggressive pharmacokinetic/pharmacodynamic (PK/PD) targets that suppress resistance and improve outcomes in severe *Pseudomonas aeruginosa* infections. A retrospective analysis of adult patients receiving CI ceftolozane/tazobactam and therapeutic drug monitoring (TDM) of both compounds was performed. Population PK/PD modeling identified the most accurate method for estimating ceftolozane/tazobactam clearance based on kidney function and Monte Carlo simulations investigated the relationship between various CI dosing regimens and aggressive PK/PD target attainment of ceftolozane/tazobactam. The 2021 non-race-based Chronic Kidney Disease Epidemiological Collaboration (CKD-EPI) equation with body surface area indexation provided the most reliable clearance estimates. Simulations showed that CI regimens of 4–6 g/2–3 g daily achieved optimal target attainment (≥90%) across all kidney function strata for MICs up to the European Committee on Antimicrobial Susceptibility Testing (EUCAST) breakpoint. Cumulative fraction of responses remained robust against key resistance phenotypes: MDR (12.6%; 83.8%), pan-beta-lactam-nonsusceptible (8.2%; 78.2%), and difficult-to-treat resistant (6.7%; 71.7%). Even among isolates with MIC >4 mg/L, aggressive target attainment was reached in 15%–40% of cases. This study suggests that CI ceftolozane/tazobactam, informed by TDM and optimized for aggressive PK/PD targets, offers a promising strategy to maximize efficacy and suppress resistance in severe *Pseudomonas aeruginosa* infections. These findings warrant prospective clinical trials of CI-based, exposure-guided therapy.

## INTRODUCTION

Antibiotic resistance is a grand challenge that threatens the foundation of modern medicine. As the effectiveness of our existing antimicrobial arsenal dwindles, preserving the utility of available agents requires a proactive, precision-based approach. Tailoring antibiotic dosing to the pathogen, site of infection, and patient-specific pharmacologic parameters is no longer optional; it is essential to prevent the selection and amplification of resistant subpopulations during treatment. Nowhere is this more critical than in the management of *Pseudomonas aeruginosa* infections, a pathogen consistently ranked among the most formidable due to its intrinsic resistance mechanisms and propensity to acquire new ones, even in the setting of guideline-concordant therapy (1).

Current antibiotic dosing recommendations are anchored in achieving clinical efficacy and minimizing toxicity but rarely account for the suppression of resistance as a core objective. This disconnect between regulatory paradigms and the clinical microbiologic reality is increasingly untenable. In a recent multicenter study conducted across 28 U.S. hospitals, resistance emerged in approximately 20% of patients with multidrug-resistant (MDR) *P. aeruginosa* infections treated with either ceftolozane/tazobactam or ceftazidime/avibactam (2). These findings underscore the urgent need for a paradigm shift from conventional dosing toward strategies that emphasize aggressive attainment of pharmacokinetic/pharmacodynamic (PK/PD) targets known to suppress resistance. For beta-lactams, achieving trough concentrations that are ≥3.8 times the MIC breakpoint has been shown to prevent bacterial resistance emergence *in vitro* with meropenem, cefepime, and ceftazidime (3).

Our recent work has extended this finding to the clinical setting and shown that exposure-optimized dosing of ceftazidime/avibactam, achieving concentrations that are ≥4 times the MIC, was associated with a nearly threefold reduction in resistance emergence (from 15.5% to 5.9%). This work provides a clear proof of concept for clinical PK/PD-driven dose redesign (4). A similar blueprint is urgently needed for ceftolozane/tazobactam because it has demonstrated superior microbiologic activity against *P. aeruginosa* compared to ceftazidime/avibactam (5). However, clinical outcomes remain sobering: 30-day all-cause mortality hovers around 20%, irrespective of the agent used at current doses (6). This suggests that optimizing drug exposure and not merely selecting the “better” antibiotic is critical to improving both microbiologic and patient-centered outcomes.

This paper presents a population PK/PD-based framework to redefine ceftolozane/tazobactam dosing through continuous infusion (CI) strategies that aim to prevent resistance emergence while maximizing therapeutic success against MDR *P. aeruginosa*. We leverage clinical data from an institution with high rates of MDR *P. aeruginosa*, where use of CI to deliver ceftolozane/tazobactam coupled with therapeutic drug monitoring (TDM) to target concentrations that are ≥4 times the MIC is the standard of care. By challenging the exposure frontier rather than the regulatory floor, we aim to shift the therapeutic trajectory from containment to prevention of antibiotic resistance for this World Health Organization high-priority pathogen.

## RESULTS

A total of 88 patients contributed 171 samples that were assayed to determine steady-state plasma concentrations of ceftolozane (Css_TOL_). Demographic and clinical data are summarized in Table 1. Most patients were males (48/88, 54.5%). Median (min-max) age, weight, and estimated glomerular filtration rate (eGFR, by the 2021 CKD-EPI formula) were 65.5 (19–90) years, 72.5 (37–119) kg, and 75.1 (4.5–165.8) mL/min/1.73 m^2^, respectively. Median (IQR) sequential organ failure assessment (SOFA) score on the day of TDM assessment was 7 (3–9). Hospital-acquired or ventilator-associated pneumonia and bloodstream infections accounted for most ceftolozane/tazobactam treatments (50/88, 56.8%). Median (min-max) length of treatment and number of TDM assessments were 10 (2–94) days and 1 (1–6), respectively. The median (min-max) Css_TOL_ was 42.9 (2.1–216.8) mg/L.

**TABLE 1:** Patients’ characteristics (*n* = 88)^*a*^

Demographic and clinical data			
Age (years)			65.5 (58.0–74.3)
Gender (male/female)			(48/40)
Body weight (kg)			72.5 (64.8–81.5)
BMI (kg/m^2^)			25.5 (23.2–28.6)
eGFR (mL/min/1.73 m^2^)			75.1 (32.5–105.7)
Augmented renal clearance			1 (1.1)
Mechanical ventilation			39 (44.3)
Vasopressor requirements			27 (30.7)
Type of infection			
Hospital- or ventilator-acquired pneumonia			32 (36.4)
Bloodstream infection			18 (20.5)
Skin and soft tissue infection			8 (9.1)
Urinary tract infection			6 (6.8)
Bone and joint infection			4 (4.5)
Malignant otitis externa			3 (3.4)
Intra-abdominal infection			2 (2.3)
Infective endocarditis			1 (1.1)
Empirical treatment			14 (15.9)
Ceftolozane/tazobactam treatment			
Median dose (g/daily)			4/2 (2/1–6/3)
Length of treatment (days)			10 (8–14.3)
N. of TDM assessment per patient			1 (1, 2)
Ceftolozane Css (mg/L)			42.9 (25.1–63.9)
Tazobactam Css (mg/L)			8.5 (4.9–15.8)
Ceftolozane:tazobactam ratio			4.6 (3.4–6.2)

^*a*^ Data are presented as median (IQR) for continuous variables, and as number (%) for dichotomous variables.

Table S1 shows the pharmacokinetic parameter estimates of the base population pharmacokinetic model, including fixed volume of distribution (V1) value and the proportional error models for residual variability. Table S2 shows the results of univariate analysis to identify significant covariates on ceftolozane clearance (CL_TOL_). Serum creatinine, serum urea, and eGFR estimated by different kidney function equations were significantly associated with CL_TOL_. Comparison of model performances in terms of Akaike information criteria (AIC) reduction after inclusion of eGFR as a covariate on CL_TOL_ is reported in Table S3. Among the eGFR equations tested, the 2021 non-race-based CKD-EPI equation with body surface area (BSA) indexation based performed better and was included in the final model. No other significant covariate was identified. Model diagnostic plots are summarized in Fig. S1 to S5. The summary of the final population pharmacokinetic model estimates is reported in Table 2. Median (min-max) individual estimate of total CL_TOL_ was 3.69 (0.45–11.3) L/h.

**TABLE 2:** Summary of the final population pharmacokinetic model^*a*^^,*b*,*c*^

Parameter	Value	RSE (%)	Shrinkage (%)
Fixed Effects			
V1	32.1	–	75.9
CL_TOL_	3.08	7.2	15.45
β_CL_TOL_	0.71	13.9	–
SD (CV%) of the random effects			
ω CL_TOL_	0.58 (62.65)	10.3	–
ω V1	0.42 (43.92)	–	–
Error model parameters			
a	12.36	9.4	–
b	0.21	15.5	–

^*a*^ V1 is the volume of central compartment for ceftolozane; CLTOL is ceftolozane clearance, β_CLTOL is the coefficient of ceftolozane CL as a function of eGFR; eGFR is the estimated glomerular filtration rate based on the 2021 chronic kidney disease epidemiology equation; and SD is the standard deviation.

^*b*^ The formulas can be represented as CLTOL = 3.08 × (eGFR/100)^0.71^.

^*c*^ –, indicates data not reported.

**Fig 1:**
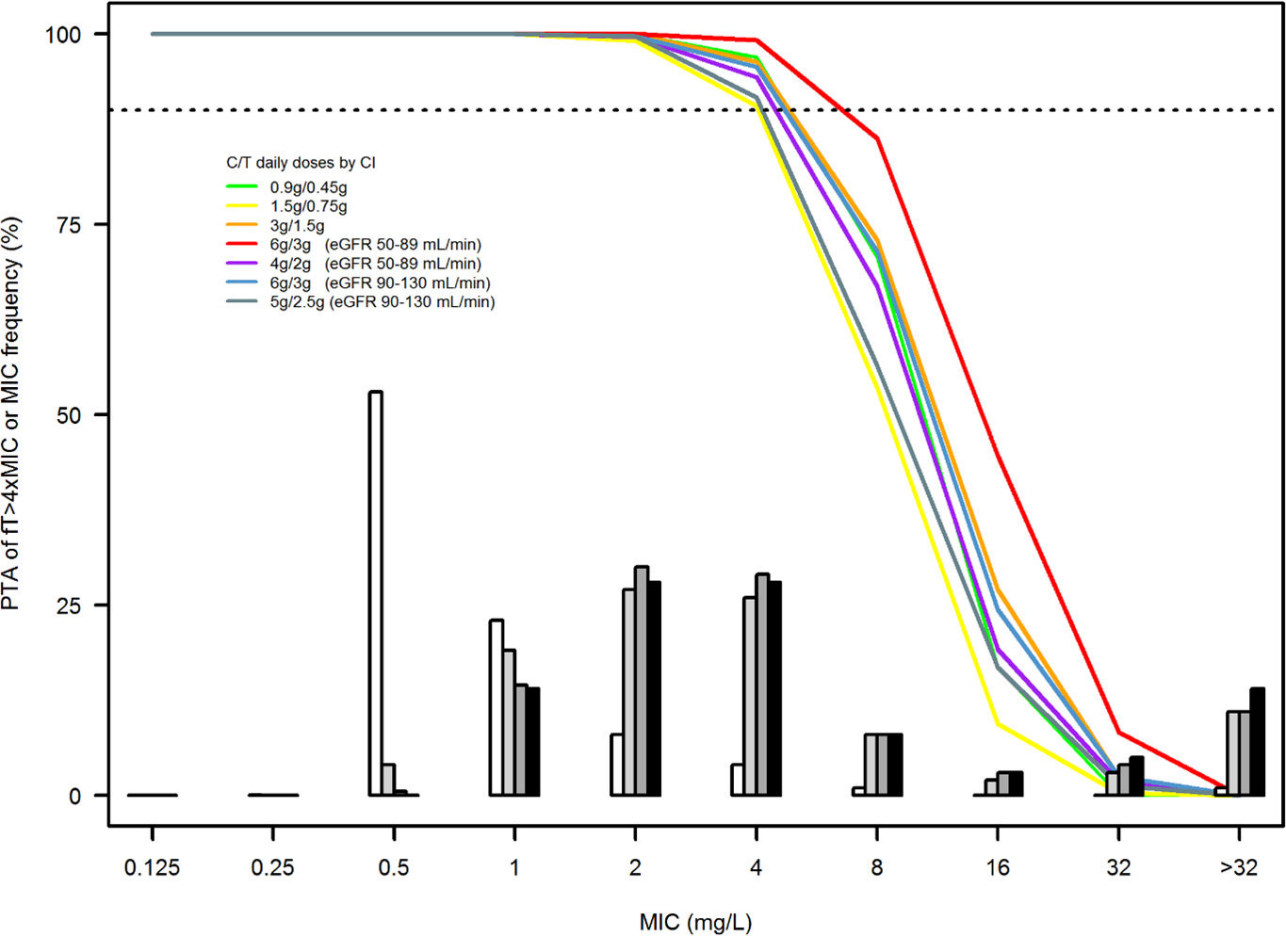
Probability of target attainment (PTA%) of achieving a ceftolozane *fCss*/MIC value of >4 with seven different dosing regimens of ceftolozane/tazobactam (0.9 g/0.45 g for eGFR <15 mL/min; 1.5 g/0.75 g mg for eGFR 15–29 mL/min; 3 g/1.5 g for eGFR 30–49 mL/min; 6 g/3 g and 4 g/2 g for eGFR 50–89 mL/min; 6 g/3 g and 5 g/2.5 g for eGFR 90–130 mL/min). The histograms are the MIC distribution frequencies of *Pseudomonas aeruginosa* isolates yielded from the US SMART Surveillance Program 2018–2020 (white bars, all isolates; light gray bars, MDR isolates; dark gray bars, pan-beta-lactam-nonsusceptible isolates; black bars, DTR isolates) (10). The horizontal dotted line identifies desirable PTA (≥90%).

Figure 1 shows the probability of target attainment (PTA) associated with the tested CI dosing regimens of ceftolozane/tazobactam in the different classes of renal function. Noteworthy, in all of the five classes of kidney function, the tested CI dosing regimens granted optimal PTAs of aggressive PK/PD target against *P. aeruginosa* strains having an MIC up to the EUCAST clinical breakpoint of 4 mg/L. It is worth noting that in the eGFR classes of 50–89 and 90–130 mL/min, this result was achievable with lower CI daily doses as 4 g/2 g daily and 5 g/2.5 g daily, respectively, compared withthe labeled 1-h infusion dose of 2 g/1 g every 8 h (6 g/3 g daily). For isolates with MIC >4 mg/L, the current approved dosage of 6 g/3 g daily by CI may not be appropriate to achieve optimal PTAs. PTAs for a *f*Css/MIC ≥1 were reported in Fig. S7.

The cumulative fraction of response (CFR) against the selected collection of *P. aeruginosa* strains associated with the tested CI ceftolozane/tazobactam dosages across the different eGFR classes is reported in Table 3. The CFR was optimal when considering all isolates as a whole (irrespective of resistance grouping), and almost always quasi-optimal when managing MDR isolates. In contrast, the CFR was suboptimal against both pan-beta-lactam nonsusceptible isolates and difficult-to-treat (DTR) isolates but was estimated to achieve this stringent target in >70% of such cases. Interestingly, by taking into account only the proportion of *P. aeruginosa* strains defined as *in vitro* resistant to ceftolozane/tazobactam according to the EUCAST (namely having MIC values > 4 mg/L), the tested dosages allowed CFR ranging around 30%–40% against all isolates as a whole, 20%–30% against MDR isolates and pan-beta-lactam non-susceptible isolates, and 15%–30% against DTR isolates (Table 4).

**TABLE 3:** Cumulative fraction of response (CFR, %) of ceftolozane/tazobactam associated with the tested continuous infusion ceftolozane/tazobactam dosages across the different eGFR classes in patients approved dosages delivered by continuous infusion for aggressive PK/PD target attainment (*f*Css_TOL_/MIC ratio ≥4) in relation to eGFR classes against four distribution types of *Pseudomonas aeruginosa* isolates from the US SMART Surveillance Program (10)^*a*^

eGFR (mL/min)	Dose (g/daily by CI)	All isolates (*n* =2531)	MDR isolates (*n* = 319)	Pan-beta-lactam nonsusceptible isolates (*n* = 207)	DTR isolates (*n* = 169)
<15	0.9/0.45	98.6	81.1	79.2	75.3
15–29	1.5/0.75	98.1	78.0	75.6	71.7
30–49	3/1.5	98.6	81.5	79.7	75.7
50–89	4/2	98.5	80.2	78.2	74.3
	6/3	98.8	83.8	82.3	78.4
90–130	5/2.5	98.2	78.6	76.5	72.6
	6/3	98.5	81.1	79.2	75.3

^*a*^ Gray shading denote cumulative fraction of response ≥90%.

**TABLE 4:** Cumulative fraction of response (CFR, %) of ceftolozane/tazobactam approved and suggested dosages for aggressive PK/PD target attainment (*f*Css_TOL_/MIC ratio ≥4) in relation to eGFR classes against *Pseudomonas aeruginosa* resistant isolates yielded from the US SMART Surveillance Program (10)

eGFR (mL/min)	Dose (mg/daily by CI)	All isolates having TOL/TAZ MIC >4 mg/L	MDR isolates having TOL/TAZ MIC >4 mg/L	Pan-beta-lactam nonsusceptible isolates having TOL/TAZ MIC >4 mg/L	DTR isolates having TOL/TAZ MIC >4 mg/L
<15	0.9/0.45	35.4	24.9	23.7	20.6
15–29	1.5/0.75	26.8	18.7	17.6	15.3
30–49	3/1.5	36.5	26.8	25.9	22.5
50–89	4/2	33.5	24.1	23.1	20.1
	6/3	43.2	33.5	32.9	28.9
90–130	5/2.5	28.2	20.4	19.5	16.9
	6/3	35.7	26.1	25.2	21.9

## DISCUSSION

 *Pseudomonas aeruginosa* is a formidable bacterial pathogen that requires optimized antibiotic exposure to improve both microbiologic and patient-centered outcomes. This clinical evaluation was performed through use of CI at ceftolozane/tazobactam dosing coupled with PK/PD modeling and simulation of patients with suspected or confirmed *P. aeruginosa* infections. Our goal was to identify dosing strategies capable of achieving aggressive, resistance-suppressive PK/PD targets. Our findings highlight the potential of CI-based regimens, especially when paired with TDM to transform the clinical utility of ceftolozane/tazobactam from mere salvage therapy to a resistance prevention tool.

 The final population PK model identified eGFR, particularly the 2021 CKD-EPI equation with BSA indexation, as the primary covariate explaining interindividual variability in CL_TOL_. The population pharmacokinetic estimates of CL_TOL_ of our model are reliable because the projected CL across eGFR lines up with the model used to justify the current labeled dosing regimen (7). Specifically, Zhanget al. identified the CL_TOL_ = 4.84 × (eGFR/100)^0.701^, while this study identified it to be 3.08 × (eGFR/100)^0.71^, that equate to central tendency estimates of CL_TOL_ that are 2.98 and 2.71 L/h, respectively. When projecting out to high kidney function (150 mL/min), the product label justification model and our current model predict values of 6.43 and 5.90 L/h, respectively. However, even with kidney function incorporated, the unexplained variability in CL_TOL_ remained substantial (62%), underscoring the limitations of relying solely on kidney function-based dosing. These findings reinforce the necessity of TDM to optimize exposure in real-time, particularly in critically ill patients where altered physiology can decouple kidney function from true drug clearance.

Most importantly, we demonstrate the feasibility of achieving an aggressive PK/PD target of *f*Css_TOL_/MIC ≥4 across all kidney function strata using CI. We applied this aggressive PK/PD target of ceftolozane against *P. aeruginosa*, which is several fold higher than previously considered in other population PK/PD models, namely 40-100% *f*T>_1xMIC_ (8). There are several reasons for preferring an aggressive rather than a conservative PK/PD target of ceftolozane for severe *P. aeruginosa* infections. First, a recent meta-analysis showed that achieving an aggressive PK/PD target of *f*Css/MIC ratio of 4–8 with CI beta-lactams greatly improved efficacy of beta-lactams for treating Gram-negative infecs in the critically ill patients (9). Aggressive PK/PD target attainment was significantly associated with better clinical cure rates (OR 1.69; 95% CI 1.15–2.49; *P* = 0.007), and a lower risk of beta-lactam resistance development (OR 0.06; 95% CI 0.01-0.29; *P* < 0.001) compared with conservative PK/PD target of *f*Css/MIC ratio of 1 (equivalent to 100%T > MIC). Additionally, an increased risk of microbiological failure (OR 26.08; 95% CI 8.72–77.95; *P* < 0.001) was observed when this target was not met. This expectation has been corroborated through a 4-year analysis of antimicrobial susceptibility testing of 751 *P*. *aeruginosa* isolates in a tertiary Italian hospital (11). This study confirmed that standard dosing of ceftolozane/tazobactam per label may cause the emergence of resistance. Furthermore, a direct link to antibiotic use and emergence of resistance has been documented. Specifically, ceftolozane/tazobactam was unavailable for use between December 2020 and May 2022 due to a manufacturing drug shortage in Italy. This allowed for a three-period analysis that identified a statistically significant (*P* < 0.001) reduction of ceftolozane/tazobactam resistance rate (25.1% reduction to 5.3%) followed by an increase (5.3% to 10.0%) with its reintroduction (11). This clearly indicates that ceftolozane/tazobactam when used at the standard 1-h infusion dosages may select antimicrobial resistance. On the contrary when dosages are optimized by the use of CI and by addressing aggressive PK/PD targets, resistance rates may decrease (4). This evidence, although indirect, indicates that resistance rate may be hampered by aggressive targeting in the clinical setting.

Beyond the use and non-use of the agent as a factor, investigators have shown that dosing matters. Specifically, Tamma and coworkers hypothesized that some risk factors for the emergence of ceftolozane/tazobactam resistance may be modifiable. They studied 28 consecutive patients infected with carbapenem-resistant *P. aeruginosa* isolates initially susceptible to ceftolozane/tazobactam. Resistance to ceftolozane/tazobactam after at least 72 h of treatment occurred in 50% of cases based on a definition of a fourfold increase in ceftolozane/tazobactam MICs from baseline. Inadequate source control increased the probability (29% vs 0%, *P* = 0.04), while the use of an extended 3-h infusion lowered the probability of resistance emergence (0% vs 29%, *P* = 0.04). Their study supports the principle that extending infusions may be protective against resistance development to ceftolozane/tazobactam (12).

Previous preclinical and clinical studies further strengthen this argument for ceftolozane/tazobactam delivery by CI. A hollow-fiber infection model against extensively drug-resistant (XDR) *P. aeruginosa* isolates comparatively assessed the efficacy of decreased ceftolozane/tazobactam doses by CI (4 g/2 g daily) versus standard doses by either extended (2 g/1 g q8h over 3 h) or intermittent infusion (2 g/1 g q8h over 1 h) (13). Noteworthy, 4 g/2 g daily by CI granted the largest bacterial density decrease and was the only regimen ensuring bactericidal effect (namely >3log10 CFU/mL decrease) against all isolates, regardless of whether the isolate was susceptible (MIC of 2 mg/L) or resistant (MIC of 8–16 mg/L) to ceftolozane/tazobactam. In line with these findings, we recently showed, by means of a pre-post quasi-experimental study of CI ceftolozane/tazobactam monotherapy for treating severe documented *P. aeruginosa* infections, that a TDM-guided approach (namely, the intervention) enabled attaining aggressive PK/PD target in all cases by using lower daily dosing regimen compared with the pre-intervention phase (3 g/1.5 g versus 6 g/3 g; *P* = 0.06), and without negatively impacting on efficacy (in terms of microbiological eradication, 30-day resistance to ceftolozane/tazobactam, clinical cure, and 30-day mortality rate) (4). Moreover, attaining an aggressive PK/PD target of *f*Css/MIC ≥4 in all the patients was associated with a reduction in ceftolozane/tazobactam resistance rate from 18.8% (pre-interventional phase) to 10.8% (post-interventional phase). As the prevalence of the post-interventional phase of 10.8% is almost halved compared with that reported by the CACTUS study (2) of 22% this may support the role of aggressive PKPD target attainment in reducing resistance development. Consequently, it was hypothesized that administering by CI the standard licensed dose for pneumonia, namely 6 g/3 g daily, could have been helpful in attaining aggressive PK/PD targets even against *P. aeruginosa* strains with MIC values up to at least 8–16 mg/L.

Our Monte Carlo simulations effectively confirmed that, by delivering the licensed dosing regimens of ceftolozane/tazobactam by CI, the PTA of aggressive PK/PD target may be optimal in all of the classes of kidney function against *P. aeruginosa* strains with an MIC up to the EUCAST clinical breakpoint of susceptibility, namely 4 mg/L. Noteworthy, when considering the eGFR classes of >50 mL/min for which a full dosage of 2 g/1 g q8h by intermittent infusion is currently recommended, the analysis showed that optimal PTA of aggressive PK/PD target may also be achieved even by delivering lower doses by CI, namely 4 g/2 g daily and 5 g/2.5 g daily in the eGFR classes of 50–89 and 90–130 mL/min, respectively. This finding is particularly important in resource-limited settings and during supply chain disruptions that can lead to drug shortages, where lowering the daily dose may be necessary.

The CFR for achieving aggressive PK/PD targets was found to be optimal across all kidney function classes when tested against a large collection of over 2,000 *P*. *aeruginosa* isolates from U.S. hospitals (11, 13). Importantly, when focusing on more resistant subgroups (MDR, 12.6%), pan-beta-lactam-nonsusceptible (8.2%), and DTR-resistant (6.7%) isolates, the CFRs remained high, ranging from 71.7% to 83.8%, though below the 90% threshold for optimality.

These results highlight the potential clinical value of CI ceftolozane/tazobactam dosing, even in challenging resistance phenotypes. Notably, this approach also demonstrated the ability to achieve with continuous infusion aggressive target exposures in approximately 15%–40% of isolates that are classified as *in vitro* resistant to ceftolozane/tazobactam (MIC >4 mg/L), as shown in Table 4. This underscores the critical role of TDM and individualized dosing in optimizing outcomes, particularly for patients infected with resistant pathogens.

 This study has several limitations. Its retrospective design limits causal inference. Only total plasma concentrations were measured; free concentrations were estimated using fixed protein binding assumptions, which may not reflect variability in critically ill patients. Although our model identified eGFR as a key covariate, substantial unexplained interindividual variability remained. The Monte Carlo simulations, while informative, rely on assumptions that may not fully capture real-world variability in pathogen behavior or host response. Additionally, our isolate set, though large and diverse, may not reflect global resistance patterns. Clinical outcomes such as time to cure or resistance emergence during therapy were not reported. However, the present study includes the same patients with assessable clinical/microbiological outcomes evaluated in the post-interventional phase of the previous study of ours mentioned above (4). Despite these limitations, the use of real-time TDM, a robust population PK model, and integration of U.S. surveillance data enhance the clinical relevance and generalizability of our findings.

  In conclusion, the findings of this population PK/PD study suggest that this innovative CI ceftolozane/tazobactam dosing strategy, by optimizing both aggressive PK/PD target attainment and CFR against *P. aeruginosa*, may have the potential of contributing meaningfully in either maximizing clinical/microbiological outcome of severe *P. aeruginosa* infections or minimizing resistance development to ceftolozane/tazobactam.

## MATERIALS AND METHODS

### Study design

Adult patients admitted in the period Nov 2023–Oct 2024 to the IRCCS, Azienda Ospedaliero-Universitaria di Bologna, Italy, receiving ceftolozane/tazobactam for treating suspected or documented *P. aeruginosa* infections and undergoing real-time TDM of both ceftolozane and tazobactam concentrations were retrospectively included in this study. The local Ethics Committee approved the study. Signed informed consent was waived due to the retrospective nature of the investigation.

Ceftolozane/tazobactam treatment included a 2 g/1 g loading dose over 1 h immediately followed by a CI maintenance dose initially based on eGFR (6 g/3 g daily by CI if eGFR >50 mL/min/1.73 m^2^; 3 g/1.5 g daily by CI if eGFR of 30–50 mL/min/1.73 m^2^, 1.5 g/0.75 g daily by CI if eGFR 15–29 mL/min/1.73 m^2^ and 0.9 g/0.45 g daily by CI if eGFR <15 mL/min/1.73 m^2^) and subsequently adapted based on TDM-guided clinical pharmacology consultation, as previously described (14). Our hospital internal protocol for TDM of beta-lactams contemplates a first TDM of steady-state plasma concentrations of ceftolozane (Css_TOL_) and of tazobactam (Css_TAZ_) between days 2 and 3, followed by subsequent twice-weekly TDM assessments guided by clinical pharmacadvices’ advices up to the end of therapy.

Total Css_TOL_ and Css_TAZ_ were measured by means of a validated liquid chromatography tandem mass spectrometry method (lower limit of quantification for ceftolozane and tazobactam were 0.2 and 0.1 mg/L, respectively) (15).

The following demographic and clinical characteristics were retrieved from electronic medical records: age, gender, weight, height, serum creatinine, serum albumin, type and site of infection, bacterial clinical isolate (whenever available) with MIC value of ceftolozane/tazobactam, underlying disease(s), ceftolozane/tazobactam dose, use of vasopressors, and use of mechanical ventilation. Kidney function and alternate body size descriptors (ABSD) were estimated using several equations (Table S4) and included (i) 2021 non-race-based CKD-EPI equation with and without BSA indexation; (ii) estimated creatinine clearance (eCL_CR_) by means of the Cockcroft-Gault equation with weight and ABSD; (iii) EKFC with and without body surface area indexation. Patients with renal replacement therapies were excluded from this study.

### Population pharmacokinetic modeling

Given that only ceftolozane is independently active against *P. aeruginosa* and this activity is not dependent on tazobactam (unlike Enterobacaterales) (16), we only modeled ceftolozane plasma concentrations by means of non-linear mixed-effect modeling using the stochastic approximation expectation minimization algorithm provided by the Monolix software (version 2024R1; Lixoft, Antony, France). Since all subjects received CI ceftolozane/tazobactam administration, a one-compartment model to fit ceftolozane concentrations was created. The first step was a linear model (base model) parameterized with zero-order administration and clearance for ceftolozane (CL_TOL_) as shown in Fig. S6. Volumes of distribution of ceftolozawerewas fixed based on a prior population pharmacokinetic model used to establish the dosing recommendations for ceftolozane/tazobactam, namely 32.1 L, as explained in Fig. S6 (17). All individual parameters were considered to be log-normally distributed. Several error models (additive, proportional, or combined additive and proportional error model) were tested for residual variability. Between-occasion variability was not considered.

In the second step, the impact of different covariates such as age, body weight, and kidney function on the base model was assessed. Covariates were selected according to a forward/backward process. In the forward step, the inclusion of a covariate in the model was based on the result of the Pearson’s correlation test between each covariate and the random effect of the estimated pharmacokinetic parameter. In the backward step, the Wald test was used to test whether a specific covariate could be removed or not from the full covariate model.

### Model selection and validation

The diagnostics criteria for comparing the different models and for identifying which significant covariates had to be included in the final model were a reduction of the objective function value (OFV) >3.82 points and of both the AIC and the Bayesian Information Criteria (BIC) of at least two points. The adequacy of the different models was assessed also by considering the goodness of fit of the observed versus predicted concentrations and the relative standard error (RSE) of the pharmacokinetic parameter estimates. Visual predictive check (VPC) showing the time course of the 10th, the 50th, and the 90th percentiles of observed data overlaid to the corresponding 90% prediction intervals was used for internal validation.

### Monte Carlo simulation

One thousand-subject Monte Carlo simulations were performed using the final population pharmacokinetic model by means of Simulx 2024R1 (Lixoft, Antony, France) in order to generate different pharmacokinetic concentration-time profiles associated with different dosing regimens of CI ceftolozane/tazobactam in relation to five classes of renal function.

The simulated daily regimens of ceftolozane/tazobactam delivered by CI were the following: 5 g/2.5 g and 6 g/3 g for eGFR of 90–130 mL/min, 4 g/2 g and 6 g/3 g daily for eGFR of 50–89 mL/min; 3 g/1.5 g daily for eGFR of 30–49 mL/min; 1.5 g/0.75 g for eGFR of 15–29 mL/min, and 0.9 g/0.45 g for eGFR of <15 mL/min. Aggressive PK/PD target attainment was defined as an *f*Css/MIC ratio of ceftolozane ≥4 (4). The free fraction (*f*) of both Css_TOL_ and Css_TAZ_ was calculated by multiplying measured values with 0.81 and 0.70, respectively, to account for plasma protein binding (18). PTAs were defined as optimal if ≥90%, quasi-optimal between 80% and 90%, and suboptimal if <80%.

The CFRs achievable with the selected ceftolozane/tazobactam dosing regimens delivered by CI were calculated against the MIC distribution of a large collection of *P. aeruginosa* isolates collected in United States hospitals stratified as all isolates as a whole (*n* = 2531) and subsets of MDR (*n* = 319), pan-beta-lactam-nonsusceptible (*n* = 207), and DTR isolates (*n* = 169) (10). The CFRs were considered optimal if >90%, quasi-optimal if between 80% and 90%, and suboptimal if <80%.

## SUPPLEMENTAL MATERIAL

Supplemental material aac.01042-25-s0001.docx Fig. S1 to S7; Tables S1 to S4.

ASM does not own the copyrights to Supplemental Material that may be linked to, or accessed through, an article. The authors have granted ASM a non-exclusive, world-wide license to publish the Supplemental Material files. Please contact the corresponding author directly for reuse.
