# Supplementary material for: Targeting Pseudomonas aeruginosa resistance at the exposure frontier: a population PK/PD blueprint for ceftolozane/tazobactam continuous infusion
Source: Antimicrob Agents Chemother. 2025 Sep 22;69(11):e01042-25. doi: 10.1128/aac.01042-25 (PMC12587603; doi:10.1128/aac.01042-25)

**Targeting *Pseudomonas aeruginosa* Resistance at the Exposure Frontier: A Population PK/PD Blueprint for Ceftolozane/Tazobactam Continuous Infusion**

Pier Giorgio Cojutti, Manjunath P. Pai, Milo Gatti, Matteo Rinaldi, Tommaso Tonetti, Antonio Siniscalchi, Pierluigi Viale, Federico Pea

**Supplemental File**

**Table S1.** Parameter estimates of the base pharmacokinetic model

|  | **VALUE** | | **STOCH. APPROX.** | | | |
| --- | --- | --- | --- | --- | --- | --- |
|  |  |  | **S.E.** | **R.S.E.(%)** | **P2.5** | **P97.5** |
| **Fixed Effects** | | | | | | |
| **V1_pop** | 32.1 | |  |  |  |  |
| **Cltol_pop** | 3.21 | | 0.3 | 9.47 | 2.67 | 3.85 |
| **Standard Deviation of the Random Effects** | | | | | | |
|  | **Value** | **C.V.(%)** |  |  |  |  |
| **omega_V1** | 0.42 | 43.9 |  |  |  |  |
| **omega_Cltol** | 0.78 | 90.9 | 0.08 | 10.3 | 0.64 | 0.95 |
| **Error Model Parameters** | | | | | | |
| **a1** | 13.26 | | 1.42 | 10.7 | 10.8 | 16.3 |
| **b1** | 0.094 | | 0.03 | 30.5 | 0.05 | 0.16 |

**Table S2.** Univariate analyses to identify significant covariates on CL_TOL_

| **Cl_TOL_** | **COEFF** | **STATISTICS** | **P-VALUE** |
| --- | --- | --- | --- |
| **arc** |  | 0.09 | 7.67E-01 |
| **crrt** |  | 0.31 | 5.76E-01 |
| **gender** |  | 3.21 | 7.66E-02 |
| **icuadm** |  | 3.1 | 8.17E-02 |
| **infect** |  | 1.31 | 2.03E-01 |
| **mv** |  | 2.31 | 1.32E-01 |
| **sex** |  | 3.21 | 7.66E-02 |
| **vasopressors** |  | 8.12 | 5.46E-03 |
| **adjbw** | 0.14 | 1.26 | 2.10E-01 |
| **age** | -0.1 | -0.7 | 5.14E-01 |
| **bmi** | -0.1 | -0.5 | 6.17E-01 |
| **bsa_dd** | 0.09 | 0.85 | 3.98E-01 |
| **bsa_m** | 0.07 | 0.69 | 4.94E-01 |
| **ckdepi2021** | 0.54 | 5.99 | 4.66E-08 |
| **ckdepi2021mlmin_bsa_dd** | 0.57 | 6.4 | 7.69E-09 |
| **ckdepi2021mlmin_bsa_m** | 0.56 | 6.33 | 1.07E-08 |
| **clcr_adjbw** | 0.48 | 5.04 | 2.57E-06 |
| **clcr_dw** | 0.47 | 4.9 | 4.49E-06 |
| **clcr_ibw** | 0.49 | 5.25 | 1.09E-06 |
| **clcr_lbw** | 0.47 | 4.95 | 3.67E-06 |
| **clcr_wt** | 0.44 | 4.55 | 1.77E-05 |
| **dw** | 0.09 | 0.81 | 4.19E-01 |
| **ekfc** | 0.53 | 5.76 | 1.26E-07 |
| **ekfc_mlmin_bsa_dd** | 0.55 | 6.15 | 2.35E-08 |
| **ekfc_mlmin_bsa_m** | 0.55 | 6.08 | 3.22E-08 |
| **ht** | 0.17 | 1.55 | 1.24E-01 |
| **ibw** | 0.18 | 1.72 | 8.89E-02 |
| **lbw** | 0.16 | 1.46 | 1.49E-01 |
| **scr** | -0.5 | -5.5 | 4.09E-07 |
| **wt** | 0.05 | 0.44 | 6.63E-01 |

| **Table S3.** Comparison of different kidney function equations as covariates of CL with the best identified structural model based on the greatest reduction in the Akaike Information Criterion (AIC) value and confidence in parameter estimates | | | |
| --- | --- | --- | --- |
| Model Number | Structure, Error Model (distribution) | Estimated Parameters | AIC value |
| Base model | 1-compartment, Combined1 (normal) F, V1 and V2, | CL_TOL_ | 2849.42 |
| 1 | 1-compartment, Combined2 (normal) Fixed V1 | CL_TOL_-β_ckdepi2021^a^ | 2713.09 |
| **2** | 1-compartment, Combined2 (normal) Fixed V1 | **CL_TOL_ -β_ckdepi2021_bsa_dd^a^** | **2711.36** |
| 3 | 1-compartment, Combined2 (normal) Fixed V1 | CL_TOL_-β_ckdepi2021_bsa_m^a^ | 2712.12 |
| 4 | 1-compartment, Combined2 (normal) Fixed V1 | CL_TOL_-β_clcr_wt^a^ | 2716.41 |
| 5 | 1-compartment, Combined2 (normal) Fixed V1 | CL_TOL_-β_clcr_lbw^b^ | 2714.83 |
| 6 | 1-compartment, Combined2 (normal) Fixed V1 | CL_TOL_-β_clcr_ibw^c^ | 2713.55 |
| 7 | 1-compartment, Combined2 (normal) Fixed V1 | CL_TOL_-β_clcr_adjbw^c^ | 2714.39 |
| 8 | 1-compartment, Combined2 (normal) Fixed V1 | CL_TOL_-β_clcr_dw^c^ | 2715.86 |
| 9 | 1-compartment, Combined2 (normal) Fixed V1 | CL_TOL_-β_EKFC^c^ | 2714.76 |
| 10 | 1-compartment, Combined2 (normal) Fixed V1 | CL_TOL_-β_EKFC_bsa_dd^c^ | 2712.48 |
| 11 | 1-compartment, Combined2 (normal) Fixed V1 | CL_TOL_-β_EKFC_bsa_m^c^ | 2712.65 |
| 12 | 1-compartment, Combined2 (normal) Fixed V1 | CL_TOL_-β_ckdepi2021_bsa_dd^a^, β_Cltaz_vasopressors_1*_ | 2708.54 |
| 13 | 1-compartment, Combined2 (normal) Fixed V1 | CL_TOL_-β_ckdepi2021_bsa_dd^a^, β_Cltaz_vasopressors_1*_, β_Clcef_icuadm_1*_ | 2707.64 |

^a^(eGFR/60); ^b^(eGFR/40); ^c^(eGFR/50), *R.S.E. >69.4%

**Table S4.** Body size descriptors and kidney function estimation equations used in this study

| Descriptor | Full Name | Unit |
| --- | --- | --- |
| wt | Weight | Weight in kg |
| ht | Height | Height in cm |
| bmi | Body mass index | Computed in kg/m^2^ |
| ibw | Ideal body weight | Estimated in kg |
| adjbw | Adjusted body weight | Estimated in kg |
| dw | Dosing weight | Estimated in kg |
| lbw | Lean body weight | Estimated in kg |
| bsa_m | Body surface area using Mosteller’s adaptation | Estimated in m^2^ |
| bsa_dd | Body surface area using Dubois-DuBois equation | Estimated in m^2^ |
| clcr_wt | Creatinine clearance using the Cockcroft-Gault equation and weight | Estimated in mL/min |
| clcr_ibw | Creatinine clearance using the Cockcroft-Gault equation and ideal body weight | Estimated in mL/min |
| clcr_adjbw | Creatinine clearance using the Cockcroft-Gault equation and adjusted body weight | Estimated in mL/min |
| clcr_dw | Creatinine clearance using the Cockcroft-Gault equation and dosing weight | Estimated in mL/min |
| ckdepi2021 | Glomerular filtration rate using the Chronic Kidney Disease Epidemiology Equation without race published in 2021 | Estimated in mL/min/1.73 m^2^ |
| ckdepi2021mlmin_bsa_m | Glomerular filtration rate using the Chronic Kidney Disease Epidemiology Equation without race published in 2021 normalized to individual body surface area- Mosteller equation | Estimated in mL/min |
| ckdepi2021mlmin_bsa_dd | Glomerular filtration rate using the Chronic Kidney Disease Epidemiology Equation without race published in 2021 normalized to individual body surface area-Dubois Dubois equation | Estimated in mL/min |
| scr_q | Serum creatinine normalized values for EKFC equation | unitless |
| ekfc | European Kidney Function Consortium (EKFC) equation | Estimated in mL/min/1.73 m^2^ |
| ekfc_mlmin_bsa_m | Glomerular filtration rate using the EKFC equation without race published in 2022 normalized to individual body surface area- Mosteller equation | Estimated in mL/min |
| ekfc_mlmin_bsa_dd | Glomerular filtration rate using the EKFC equation without race published in 2022 normalized to individual body surface area- Dubois-DuBois equation | Estimated in mL/min |

**Equations**

The formulas used to compute key variables are included below based on code used in STATA

****body weight**

gen ibw = 45.5 + 2.3*((ht/2.54)-60)

replace ibw = ibw + 4.5 if sex=="Male"

gen adjbw = 0.4*(wt-ibw) + ibw

gen dw= ibw

replace dw = wt if wt<ibw

replace dw = adjbw if wt>=1.25*ibw

gen bsa_m = ((wt*ht)/3600)^0.5

gen bsa_dd = (ht^0.725)*(0.007184)* (wt)^0.425

gen lbw = (9270*wt)/(6680+216*bmi)

replace lbw = (9270*wt)/(8780+244*bmi) if sex=="Female"

****CLcr**

gen CLcr_wt = (140-age)*wt/(72*scr)

replace CLcr_wt = CLcr_wt * 0.85 if sex=="Female"

gen CLcr_ibw = (140-age)*ibw/(72*scr)

replace CLcr_ibw = CLcr_ibw * 0.85 if sex=="Female"

gen CLcr_adjbw = (140-age)*adjbw/(72*scr)

replace CLcr_adjbw = CLcr_adjbw * 0.85 if sex=="Female"

gen CLcr_dw = (140-age)*dw/(72*scr)

replace CLcr_dw = CLcr_dw * 0.85 if sex=="Female"

gen CLcr_lbw = (140-age)*lbw/(72*scr)

****GFR**

*CKD EPI 2021

gen CKDEPI2021 = 142*((scr/0.7)^-0.242)*(0.9938^age)*1.012 if sex=="Female" & scr<=0.7

replace CKDEPI2021 = 142 * ((scr/0.7)^-1.200) * (0.9938^age) * 1.012 if sex=="Female" & scr>0.7

replace CKDEPI2021 = 142 * ((scr/0.9)^-0.302) * (0.9938^age) if sex=="Male" & scr<=0.9

replace CKDEPI2021 = 142 * ((scr/0.9)^-1.200)* (0.9938^age) if sex=="Male" & scr>0.9

gen CKDEPI2021mlmin_bsa_m = CKDEPI2021*bsa_m/1.73

gen CKDEPI2021mlmin_bsa_dd = CKDEPI2021*bsa_dd/1.73

*EKFC

gen scr_q = scr/0.9 if sex=="Male"

replace scr_q = scr/0.7 if sex=="Female"

gen ekfc = 107.3*scr_q^-0.332 if age<=40 & scr_q<1 & sex=="Female"

replace ekfc = 107.3*scr_q^-1.132 if age<=40 & scr_q>=1 & sex=="Female"

replace ekfc = 107.3*scr_q^-0.332 if age<=40 & scr_q<1 & sex=="Male"

replace ekfc = 107.3*scr_q^-1.132 if age<=40 & scr_q>=1 & sex=="Male"

replace ekfc = 107.3*(scr_q^-0.332)*0.990^(age-40) if age>40 & scr_q<1

replace ekfc = 107.3*(scr_q^-1.132)*0.990^(age-40) if age>40 & scr_q>=1

*EKFC (European Kidney Function )- in ml/min

gen ekfc_mlmin_bsa_m = ekfc*bsa_m/1.73

gen ekfc_mlmin_bsa_dd = ekfc*bsa_dd/1.73

**Figure S1**. Ceftolozane population-predictions vs. observations and individual-predictions vs. observations for the final population pharmacokinetic model


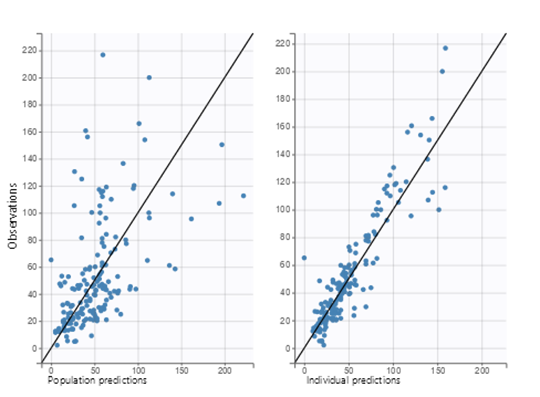


**Figure S2**. Scatter plot of the residual vs. time and of the residual vs. individual predictions of ceftolozane for the final population pharmacokinetic model

**Figure S3**. Distribution of the Individual Weighted Residuals and of the Normalized Prediction Distribution Errors of ceftolozane for the final population pharmacokinetic model

**Figure S4**. Visual predictive check (VPC) for the ceftolozane final population pharmacokinetic model

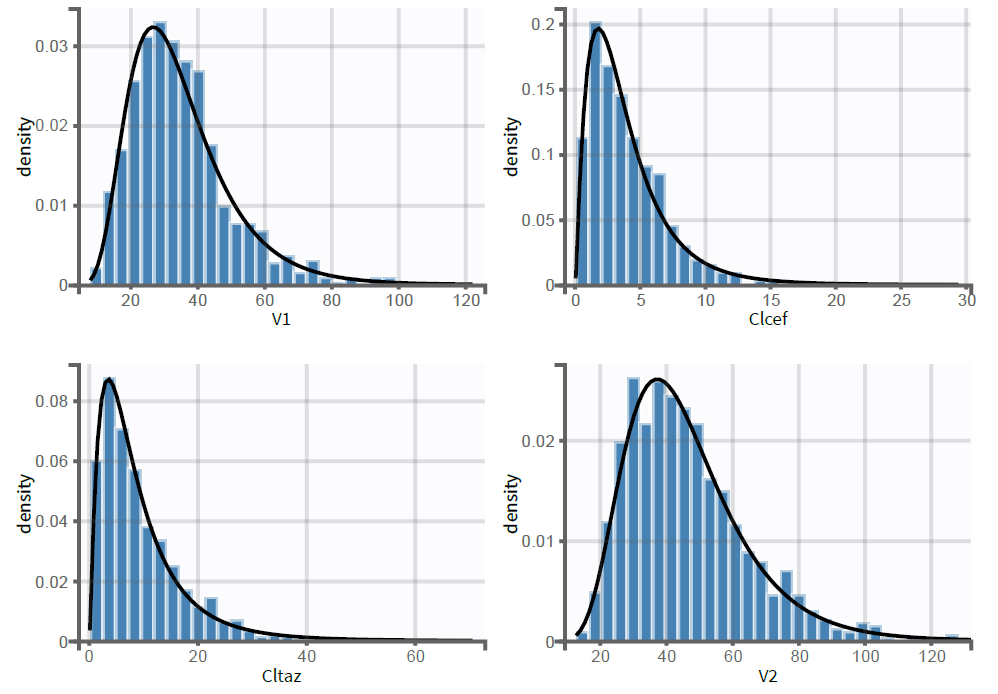
**Figure S5**. Distribution of the individual parameters

**Figure S6.** Structure of the base pharmacokinetic model for ceftolozane


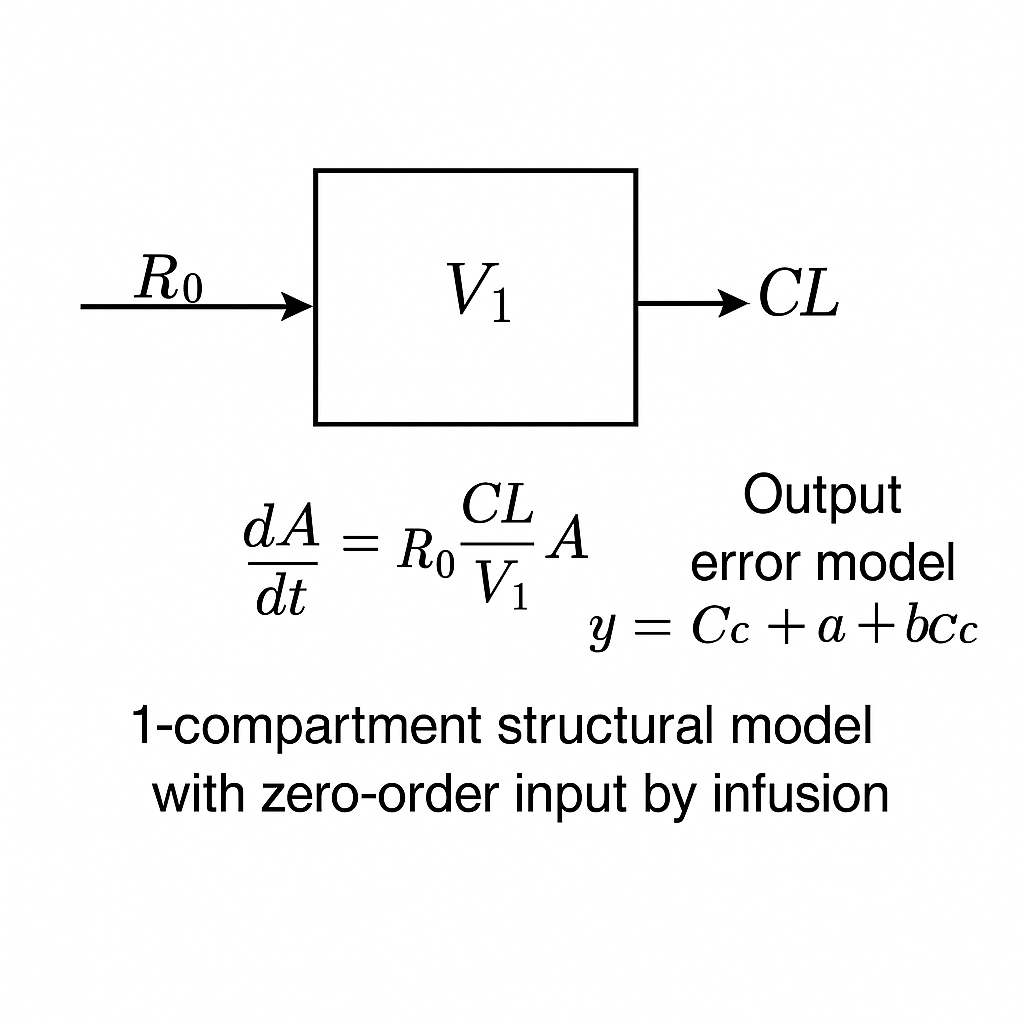


**Initial Parameter Estimates**

| Mean Parameter Estimates | Pop PK (Merck &Co, Inc)* |
| --- | --- |
| CL = CL_TOL_ (L/h) | 5.4 |
| V1 = V_TOL_ (L) | 32.1 |

*Caro L, Larson KB, Nicolau DP, De Waele J, Kuti JL, Saralaya R, Gadzicki E, Adedoyin A, Zeng Z and Rhee EG, P2225 ECCMID 21-24 April, 2018, Madrid, Spain, and study report of MK-7625A PN007 (data on file at Merck & Co., Inc., Kenilworth, New Jersey, USA). Ceftolozane/tazobactam 3000 mg (ceftolozane 2000 mg/tazobactam 1000 mg) or adjusted based on CrCL q8h by IV infusion over 1 hour in ventilated patients with suspected or confirmed pneumonia. [EUCAST rationale document Ceftolozane/tazobactam Version 1.0 Page 6 of 16]. F represented in this model was set based on the known formulation fraction of ceftolozane : tazobactam of 2:1.

**Figure S7**. Probability of target attainment (PTA%) of achieving a ceftolozane *fCss*/MIC value of >1 with seven different dosing regimens of ceftolozane/tazobactam (0.9 g/0.45 g for eGFR <15 mL/min; 1.5 g/0.75 g mg for eGFR 15-29 mL/min; 3 g/1.5 g for eGFR 30-49 mL/min; 6 g/3 g and 4 g/2 g for eGFR 50-89 mL/min; 6 g/3 g and 5 g/2.5 g for eGFR 90-130 mL/min). The histograms are the MIC distribution frequencies of *Pseudomonas aeruginosa* isolates yielded from the US SMART Surveillance Program 2018-2020 (white bars, all isolates; lightgray bars, MDR isolates; darkgray bars, pan-beta-lactam-nonsusceptible isolates; black bars, DTR isolates). The horizontal dotted line identifies desirable PTA (≥90%).


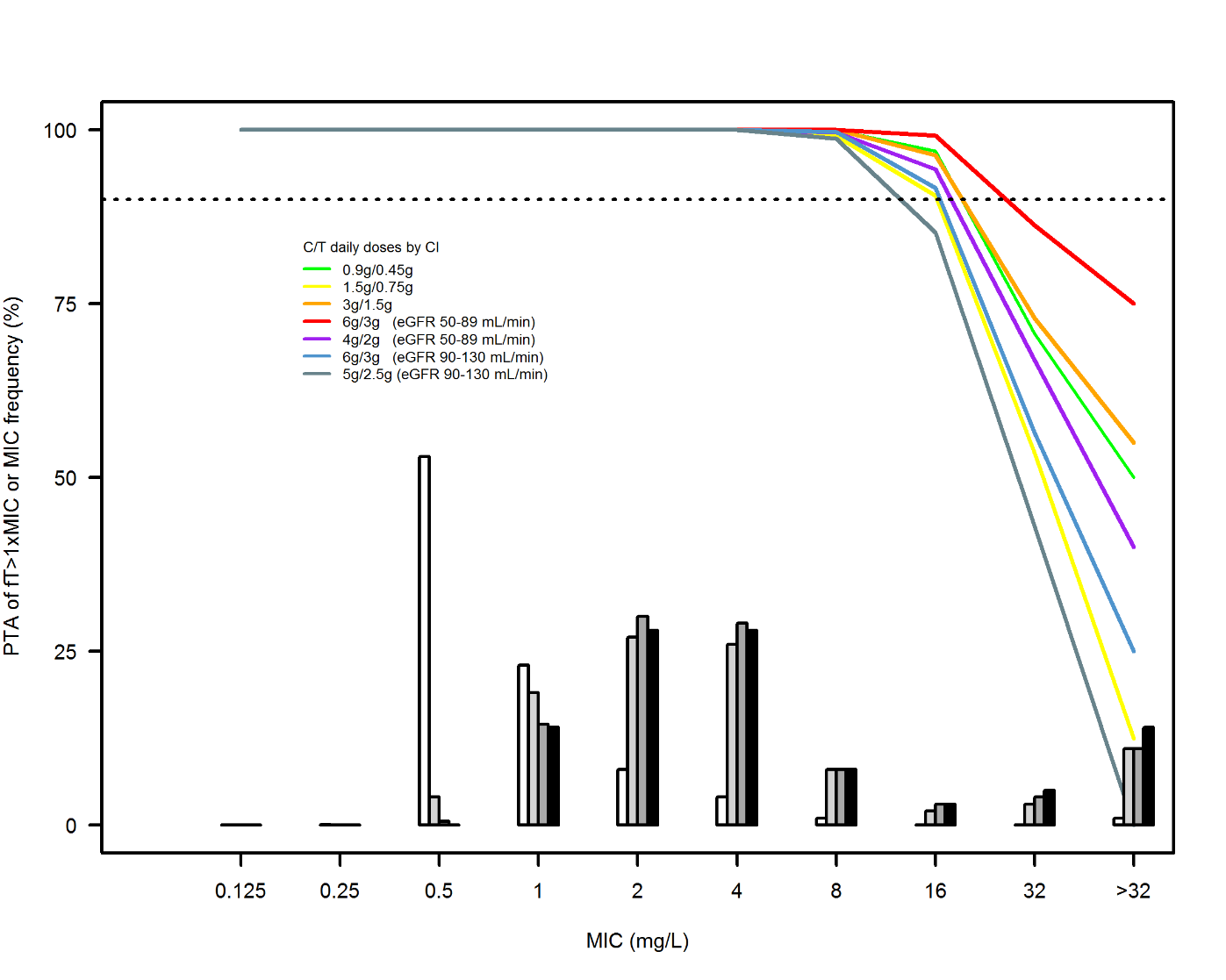

Supplement: Supplemental material — Fig. S1 to S7; Tables S1 to S4. [file aac.01042-25-s0001.docx]
